# *De novo* chromosome-level genome assembly of Chinese motherwort (*Leonurus japonicus*)

**DOI:** 10.1038/s41597-023-02901-w

**Published:** 2024-01-09

**Authors:** Xinrui Wang, Lili Zhang, Gang Yao, Xiangfeng Wang, Shu Yi, Tan Meng, Dian Meng, Weikai Chen, Li Guo

**Affiliations:** 1 Peking University Institute of Advanced Agricultural Sciences, Shandong Laboratory of Advanced Agricultural Sciences at Weifang, Weifang, Shandong 261325 China; 2 Weifang Institute of Technology, School of Modern Agriculture and Environment, Weifang, Shandong 261101 China

**Keywords:** Computational biology and bioinformatics, Plant sciences, Genome

## Abstract

Chinese motherwort (*Leonurus japonicus*), a member of Lamiaceae family, is a commonly used medicinal herb for treating obstetrical and gynecological diseases, producing over 280 officinal natural products. Due to limited genomic resources, little progress has been made in deciphering the biosynthetic pathway of valuable natural products in *L. japonicus*. Here, we *de novo* assembled the *L. japonicus* genome using high-coverage ONT long reads and Hi-C reads. The chromosome-level genome assembly contained ten chromosomes representing 99.29% of 489.34 Mb genomic sequence with a contig and scaffold N50 of 7.27 Mb and 50.86 Mb, respectively. Genome validations revealed BUSCO and LAI score of 99.2% and 21.99, respectively, suggesting high quality of genome assembly. Using transcriptomic data from various tissues, 22,531 protein-coding genes were annotated. Phylogenomic analysis of 13 angiosperm plants suggested *L. japonicus* had 58 expanded gene families functionally enriched in specialized metabolism such as diterpenoid biosynthesis. The genome assembly, annotation, and sequencing data provide resources for the elucidation of biosynthetic pathways behind natural products of pharmaceutical applications in *L. japonicus*.

## Background & Summary

*Leonurus japonicus* Houtt., known as Chinese motherwort or “*Yi Mu Cao”*, is a medicinal herb widely used in Traditional Chinese Medicine for thousands of years in China, Japan, and Korea for the treatment of obstetrical and gynecological diseases^1^. The annual plant *L. japonicus* belongs to the Lamioideae subfamily within Lamiaceae (mint) family, a highly diverse family containing more than 7,000 species^2^. To date, genome resources of 32 members in Lamiaceae have been published including some economically important species (medicinal, culinary, and fragrance usage) such as *Salvia officinalis* (sage)^3^, *Salvia bowleyana* (southern danshen)^4^, *Lavandula angustifolia* (lavender)^5^ and *Prunella vulgaris* (self heal)^6^, while in Lamioideae subfamily two genomes *Leonotis leonurus* (Lion’s tail)^6^ and *Pogostemon cablin* (Patchouli)^7^ are available. Current research on *L. japonicus* mainly focused on phytochemistry, pharmacology, and clinical applications, while little genomic resource is available besides karyotyping (2n = 2x = 20)^1,8–12^. According to *China Pharmacopoeia* (1977 to 2015 A.D.), *L. japonicus* can be used to treat menstrual disturbance, dysmenorrhea, amenorrhea, postpartum hemorrhage, postpartum lochiorrhea, invigorating blood circulation, diuretics, and dispelling edema^1,10–12^. The medicinal properties of *L. japonicus* come from its various natural products, including terpenoids, alkaloids, steroids, and flavonoids^12–14^. About 280 natural products have been identified in *L. japonicus*, notably leonurine and stachydrine, which are key in treating obstetric and gynecological conditions^1,10,15^. However, their natural yield is low in the plant^16^, making the elucidation of their biosynthetic pathways crucial for bioengineering and industrial production. This research is hindered by limited genetic resources, with only one published genome available^17^, insufficient for studying the species’ genetic diversity. Therefore, decoding the *L. japonicus* genome is essential for understanding and enhancing the production of these medicinal compounds.

**Fig. 1:**
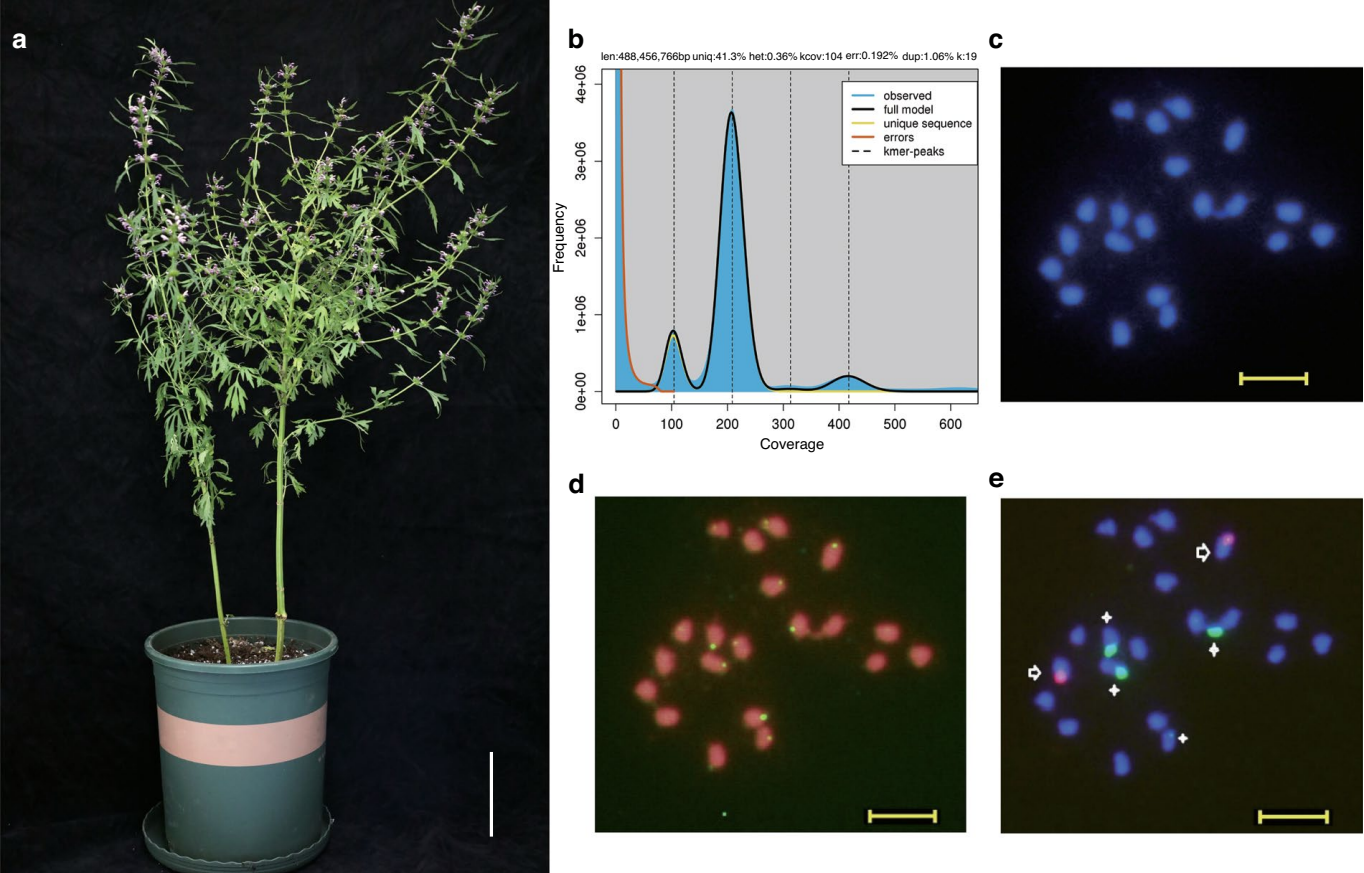
The genome survey and karyotyping of *Leonurus japonicus*. (**a**) A photograph of *L. japonicus* plant sequenced for genome assembly. Scale bar, 10 cm. (**b**) Kmer-19 histogram generated using Illumina reads. Genome size and heterozygosity rate were estimated using GenomeScope2. (**c**) Cytological analysis of *L. japonicus* chromosomes in root tip cells. The chromosomes were stained with DAPI (blue). (**d**) Karyotyping of *L. japonicus* chromosomes based on telomere-specific probes FISH signals (green). (**e**) Karyotyping of *L. japonicus* chromosomes based on 5 S rDNA (red; arrows) and 18 S rDNA (green; stars) FISH signals. Scale bar, 50 μm.

**Fig. 2:**
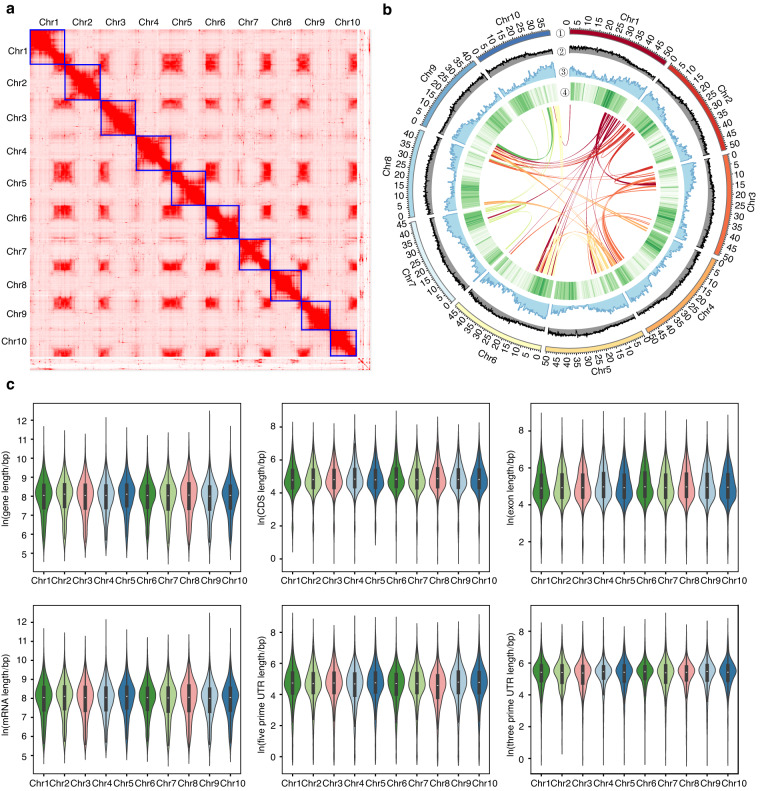
Overview of the genome assembly and annotation of *Leonurus japonicus*. (**a**) Hi-C interaction heatmap of *L. japonicus* genome assembly. (**b**) Circos plot showing the gene features at 100 kb windows across the 10 chromosomes in *L. japonicus*. From outer to inner ring: chromosome ideogram, GC content, gene density, and TE (transposable element) density. (**c**) Violin plots of genomic features, including gene length, CDS length, exon length, mRNA length, five prime UTR (untranslated region), and three prime UTR.

Here, we report a chromosome-scale genome assembly of *L. japonicus* plant (Fig. 1a) YMC-RGv1.0 using a combination of Oxford Nanopore Technology (ONT) long reads, Illumina short reads (NGS) and high-throughput chromatin conformation capture sequencing (Hi-C) reads. In total, 130.69 Gb paired-end NGS reads of 150 bp, 46.33 Gb ONT reads with N50 of 17.67 kb, and 321.46 Gb Hi-C reads for *L. japonicus* were obtained (Table 1). The k-mer (k = 19) frequency analysis revealed that *L. japonicus* had an estimated genome size of 488.46 Mb with a heterozygosity rate of 0.36% (Fig. 1b). Karyotyping of *L. japonicus* confirmed its karyotype of 2n = 2x = 20 (Fig. 1c-d) as reported previously^8^. The *L. japonicus* genome was assembled from ONT reads by *NextDenovo* and polished by NGS reads using *NextPolish*, producing a draft assembly of 173 contigs with a contig N50 of 7.27 Mb (Table 2). The contigs were then scaffolded and corrected using Hi-C data, unambiguously anchoring them onto 10 chromosomes (Fig. 2a) for *L. japonicus* with a scaffold N50 of 50.86 Mb (Table 2), representing 99.29% of total contig sequences (Table 2; Fig. 2b). The final genome (YMC-RGv1.0) was 489.34 Mb in length, close to the estimated *L. japonicus* genome size (Fig. 1b) but smaller than the 590 Mb *L. leonurus* genome, another recently published genome in Lamioideae^6^. Genome annotation of YMC-RGv1.0 predicted 22,531 protein-coding genes with an N50 of 1,536 bp using *MAKER2* pipeline, among which 22,458 were located on chromosomes (Table 2; Fig. 2c).

**Table 1:** Statistics of the sequencing data used for genome assembly of *Leonurus japonicus*.

Statistics	ONT	NGS	Hi-C	RNA-seq
Raw data (bp)	46,332,547,308	130,694,503,200	321,464,970,000	30,964,834,068
N50 (bp)	17,669	150	150	150
Longest reads (bp)	107,580	150	150	150
Mean read length (bp)	16,549	150	150	150
Coverage ( >0 X)	98.48	93.58	99.00	N/A
Mapping rate	99.97	99.27	N/A	N/A

**Table 2:** Statistics of the sequencing data used for genome assembly of *Leonurus japonicus*.

Features	Values
**Genome assembly**	
Total length (bp)	489,343,149
Number of contigs	173
Longest contig length (bp)	25,310,982
Contig N50 (bp)	7,270,386
Contig N90 (bp)	1,487,363
Number of scaffolds	46
Longest scaffold length (bp)	53,664,944
Scaffold N50 (bp)	50,856,816
Scaffold N90 (bp)	42,478,245
Anchored to chromosome (Mb, %)	485.86 (99.29%)
BUSCO (%)	99.20
LAI	21.99
**Genome annotation**	
Number of genes	22,531
No. of genes in 10 chromosomes	22,458
Repetitive sequence (%)	63.27
BUSCO (%)	94.70

In summary, the genomic resources presented in this study will be valuable to studying the biology of *L. japonicus*. The high-quality genome assembly and annotation will facilitate the dissection of biosynthesis pathways of medicinal natural products in *L. japonicus*. Also, the chromosome-scale genome of *L. japonicus* will help comparative genomic studies to illustrate the genome evolution of Lamiaceae family and beyond.

## Methods

### Sample preparation

*L. japonicus* cultivar YMC-01 was grown with a day/night cycle of 25 °C/14 h light and 18 °C/10 h dark, at a humidity of ~60% in the greenhouse of Peking University Institute of Advanced Agricultural Sciences. Young leaf tissues were sampled from a 7-week-old *L. japonicus* plant for high molecular weight (HMW) DNA extraction. Root, stem, leaf, and flower tissues at 1 day post anthesis were harvested for RNA extraction. The collected materials were frozen immediately in liquid nitrogen and stored at −80 °C. HMW DNA (N50 > 50 kb) was obtained by a modified cetyltrimethylammonium bromide (CTAB) method^18^. RNA extraction was conducted using Trizol reagent following manufacturer recommendation. The total RNA extracts with a RIN (RNA integrity number) value ≥ 7.0 were used for downstream library construction for transcriptome sequencing.

### Barcoding analysis

The extracted DNA was then used as PCR templates to amplify *rbcL* DNA barcode employing a pair of universal primers^19^ (F: 5′-AGACCTWTTTGAAGAAGGTTCWGT-3′; R: 5′-TCGGTYAGAGCRGGCATRTGCCA-3′. The PCR reaction conditions were as follows: initial denaturation at 95 °C for 5 min; then 35 cycles of 50 s at 94 °C, 30 s at 60 °C, and 1 min at 72 °C. Finally, an additional of 7 min was continued at 72 °C to complete the reaction. All reactions were carried out in SureCycler 8800 Thermal Cycler (Agilent, USA). The PCR products were electrophorized on 1% agarose gel using 1 kb DNA marker (Bioline, UK) to confirm the amplification length. The product was next sent to Tsingke Biotechnology Co., Ltd. for sequencing, and BLAST comparison was performed at National Center for Biotechnology Information (NCBI) after sequencing. For species identification, the *rbcL* sequence was identified with Barcode of Life Database (BOLD) system^20^.

### Karyotype analysis

Actively growing root tips from germinating *L. japonicus* seeds were treated in ice water at 0 °C for 24 h to accumulate metaphases before fixation in 3:1 (v/v) 100% ethanol:acetic acid. The root tips were digested with an enzyme mixture containing cellulase and pectolyase and squashed in a drop of 45% acetic acid. The chromosomes were counterstained with DAPI (2 mg·ml^−1^) and mounted in Vectashield mounting medium. Images were captured using an Olympus BX63 fluorescence microscope equipped with an Olympus DP80 CCD camera and were processed using cellSens Dimension 1.9 (Olympus Corporation). Fluorescent probes based on telomere and conservative repeated sequences of 5 S ribosomal DNA (rDNA) or 18 S rDNA are used for fluorescence *in situ* hybridization (FISH) on the samples to determine the chromosomal ploidy characteristics of the species.

### Genome and transcriptome sequencing

The NGS library was constructed using NEB Next® Ultra™ DNA Library Prep Kit for Illumina (NEB, USA) following its standard protocol. The Hi-C library was prepared from cross-linked chromatins using a standard Hi-C protocol. Both the NGS and Hi-C libraries were sequenced on Illumina NovaSeq 6000 platform to generate 150 bp paired-end reads. The Nanopore DNA library was prepared following the Ligation Sequencing SQK-LSK109 Kit (Oxford Nanopore Technologies, Oxford, UK) protocol and sequenced using Oxford Nanopore GridION (20 kb) platform. The transcriptome sequencing libraries from different tissues (root, stem, leaf, flower) were prepared using Illumina True-seq transcriptome kit (Illumina, CA). The libraries were then sequenced on Illumina NovaSeq 6000 platform to generate 150 bp paired-end reads.

### Genome assembly

The genome size and heterozygosity rate of *L. japonicus* were estimated by performing K-mer frequency analysis with Jellyfish v2.3.0^21^ and GenomeScope v2.0^22^ using NGS reads. *De novo* genome assembly using ONT long reads was carried out with NextDenovo v2.5.0 (https://github.com/Nextomics/NextDenovo). To correct sequencing errors, we polished the draft assembly for three rounds using Illumina short reads by NextPolish v1.3.1^23^. Then Hi-C sequencing data were used to anchor all contigs using the pipeline of Juicer v1.5^24^, 3D-DNA v180419^25^ and Juicebox v1.11.08^26^. Finally, we assessed the completeness of the genome assembly using Benchmarking Universal Single-Copy Orthologs (BUSCO) v4.0.6^27^ with default parameters. The assembly continuity was also evaluated by calculating the Long Terminal Repeat Assembly Index (LAI) using LTR_retriever^28^ with default parameters.

### Genome annotation

For the annotation of repetitive elements, we implemented a hybrid strategy that entailed both *de novo* prediction and homology-based searches. RepeatModeler2 v2.0.1^29^ was used to build a *de novo* repeat library. Subsequently, we annotated and masked the assembled genome using RepeatMasker v4.10 software^30^. For gene annotation, transcriptomic reads from different tissues (root, leaf, stem and flower) were first assembled using StringTie^31^. The protein sequences used for homology-based prediction were from *S. baicalensis*^32^ and universal Swiss-Prot proteins^33^. Then, we combined transcriptomic assemblies, *ab initio* prediction, and homolog protein mapping with MAKER2^34^ to predict gene structures. Finally, only the gene sets with Annotation Edit Distance (AED) lower than 0.5 were retained for further study. The BUSCO completeness of predicted gene models was assessed against eudicots_odb10 datasets under the protein mode. Gene functions were identified using NCBI protein database with an *E*-value threshold of 1e^−10^ for BLAST searches and annotated using eggNOG-mapper v2^35^ database.

### Gene family identification and Phylogenetic analysis

**Fig. 3:**
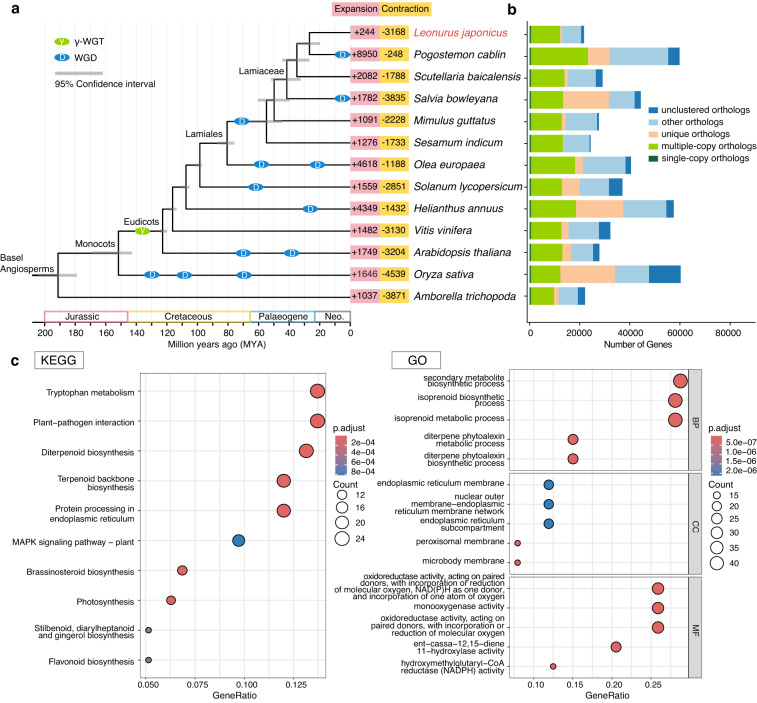
*Leonurus japonicus* genome evolution. (**a**) Phylogenetic tree with 51 single-copy orthologs from 13 species identified by Orthofinder to show divergence times. (**b**) Gene distribution in *L. japonicus* and 12 other representative species. (**c**) KEGG and GO enrichment analysis of *L. japonicus*-expanded genes.

Orthologs and orthogroups among *L. japonicus* and 12 other genomes (Table 3, Fig. 3a) were identified using the software OrthoFinder v2.5.4^36^ with default values setting and ‘-M msa’ activated. The longest predicted protein of each gene was used as the representative input for the OrthoFinder analysis. Then we removed poorly aligned regions of multiple protein sequence alignments with TrimAl v1.4.1^37^. RAxML v8.2.12^38^ was used to a build Maximum Likelihood (ML) phylogenetic tree using the GAMMAJTT model, with *A. trichopoda* as an outgroup. The CodeML and MCMCTree programs in the PAML v4.9^39^ were used to analyze amino acid substitution models and estimate divergence times. Five calibration points (*S. indicum* vs. *O. europaea*: 75.8~93.9 MYA, *O. europaea* vs. *S. lycopersicum*: 97.5~107.3 MYA, *V. vinifera* vs. *A. thaliana*: 109.0~123.5 MYA, *A. thaliana* vs. *O. sativa*: 143.0~174.8 MYA, and *O. sativa* vs. *A. trichopoda*: 179.9~205.0 MYA) derived from the TimeTree database^40^ were applied to constrain the divergence times of the nodes. Gene families that underwent expansion or contraction events were identified by CAFE5^41^ software. The identified expanded gene families of *L. japonicus* were then subjected to further analysis of Gene Ontology (GO) term enrichment^42^ and Kyoto Encyclopedia of Genes and Genomes (KEGG) enrichment^43^, and the *p*-value of significant enrichment was set as 0.05 in GO term and KEGG enrichment analysis. The gene distribution results showed that 184 gene families (573 genes) were unique to *L. japonicus* compared with other plants (Fig. 3b). *L. japonicus* genome had 58 expanded gene families (463 genes) that were enriched in pathways such as tryptophan metabolism, diterpenoid biosynthesis, and plant-pathogen interaction (Fig. 3a,c).

**Table 3:** Information and sources of sequences of 13 species in the phylogenetic tree.

Species	Database	Accession number
*Leonurus japonicus*	This study	This study
*Arabidopsis thaliana*	JGI	Araport11
*Amborella trichopoda*	JGI	Amborella trichopoda var. SantaCruz_75 HAP1 v2.1
*Helianthus annuus*	NCBI	GCF_002127325.2
*Mimulus guttatus*	NCBI	GCF_000504015.1
*Olea europaea*	NCBI	GCF_002742605.1
*Oryza sativa*	RIGW	MH63RS3
*Pogostemon cablin*	GWH	GWHBAZF00000000
*Scutellaria baicalensis*	GWH	GWHAOTC00000000
*Salvia bowleyana*	GWH	GWHASIU00000000
*Sesamum indicum*	NCBI	GCF_000512975.1
*Solanum lycopersicum*	SolOmics	SL5.0
*Vitis vinifera*	JGI	Vitis vinifera v2.1

### Synteny analysis

**Fig. 4:**
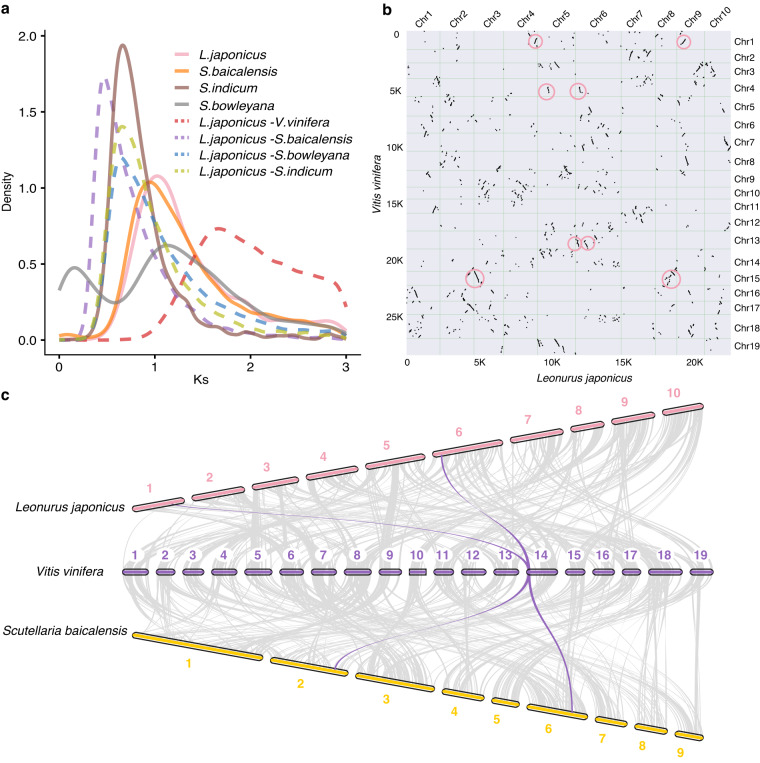
Shared WGD event of Lamiales. (**a**) Distribution of *Ks* (synonymous substitution rate) for gene pairs in syntenic blocks from intraspecies or interspecies genome comparison involving *L. japonicus* and different angiosperm species. (**b**) Homologous dot plot between *L. japonicus* and *V. vinifera* chromosomes. (**c**) Genome synteny among *L. japonicus*, *S. baicalensis*, and *V. vinifera*.

The syntenic analysis was performed by JCVI v1.1.19^44^. We identified synteny blocks by performing an all-against-all BLAST search and chaining the hits with a distance cutoff of 20 genes. Additionally, we required each synteny block to have at least five gene pairs. To estimate the timing of the WGD event in *L. japonicus*, *Ks* values of *L. japonicus* syntenic block genes were calculated using ParaAT v2.0^45^. Our analysis indicated a significant WGD in *L. japonicus*, with a prominent *Ks* peak at 1.03 (Fig. 4a). Compared with *Vitis vinifera*, which lacks genome duplication post-γ event^46^, both *L. japonicus* and *S. baicalensis* exhibit a 2:1 collinearity relationship with grape, highlighting the WGD’s significance (Fig. 4b,c). The WGD, estimated to have occurred around 72 MYA based on the *Ks* distribution and mutation rate, appears widespread in Lamiales genomes (Fig. 3a), with exceptions like the Oleaceae family^3,47^.

## Data Records

The sequencing dataset was deposited in public repositories. Illumina, Oxford Nanopore, Hi-C, and RNA-seq sequencing data used for genome assembly and annotation have been deposited in the NCBI Sequence Read Archive (SRA) database with accession numbers SRR25110886^48^, SRR25110887^49^, SRR25110885^50^, SRR25110888^51^, SRR25110889^52^, SRR25110890^53^ and SRR26458975^54^, respectively, under the BioProject accession number PRJNA989396. The chromosomal-level genome assembly was deposited in the GenBank database of NCBI with accession number GCA_030762865.1^55^. Moreover, the genome annotation, multiple sequence alignment, and gene family expansion files in phylogenetic analysis have been deposited at the Figshare database^56^.

## Technical Validation

### Plant Identification

The *ribulose 1,5-biphosphate carboxylase (rbcL)* gene in the chloroplast genome has been explored as a DNA barcode for medicinal plant identification^57,58^. The size of *rbcL* amplicon by a universal primer pair was 895 bp. The partial sequence of *rbcL* was obtained and deposited into GenBank with the accession number OR790520. The BOLD system and NCBI were used to BLAST the sequence, and the results showed that the plant material was *L. japonicus*.

### Genome assembly validation

The quality of the assembly YMC-RGv1.0 was evaluated using four approaches. Firstly, comparing the final genome assembly size with the estimated size, the results showed that they were both close to 489 Mb (Table 2; Fig. 1b). The Hi-C heatmap for our *L. japonicus* genome assembly revealed 10 chromosome models (Fig. 2a), consistent with our karyotype analysis (2n = 20) (Fig. 1c–e). Secondly, BUSCO analysis found 99.20% eudicot BUSCOs in the *L. japonicus* genome, and 94.70% eudicot BUSCOs in its predicted gene models (Table 2). Thirdly, LAI score (21.99) met the quality standard for reference genomes (LAI > 20) (Table 2). Finally, sequencing data were mapped to the genome using SAMtools v1.16.1^59^ to verify the accuracy, which showed 98.48% genome coverage and mapping rates of 99.97% for ONT data, and 93.58% genome coverage and 99.27% for NGS data (Table 1). These results indicated that the *L. japonicus* assembly was of high accuracy and completeness.

## Data Availability

No custom code was used for this study. All data analyses were conducted using published bioinformatics software with default settings unless otherwise specified.
